# COVID-19 Testing Crisis Management Through a Public-Private Partnership in Sindh, Pakistan

**DOI:** 10.9745/GHSP-D-21-00308

**Published:** 2022-02-28

**Authors:** Saba Jamal, Javeria Aijaz, Najam Shah, Fouzia Naseer, Maimoona Khan, Muzaffar Ali Odho, Abdul Bari Khan

**Affiliations:** a Indus Hospital & Health Network, Karachi, Pakistan.; b Health Department, Government of Sindh, Karachi, Pakistan.

## Abstract

As the coronavirus disease (COVID-19) pandemic spread, meeting the testing needs to control the spread of infection became a major challenge worldwide. In Pakistan, the lack of the requisite infrastructure and training compounded the acute shortage of testing kits and other consumables. Against this backdrop and to urgently improve province-wide access to high-quality COVID-19 polymerase chain reaction (PCR) testing with rapid turnaround times, the Government of the Sindh (GoS) province of Pakistan entered into a public-private partnership with Indus Hospital & Health Network (IHHN). Under this partnership, the GoS undertook sample collection and Indus Hospital in Karachi, Sindh, centralized testing. We describe the implementation strategies adopted by the partnership, as well as the challenges, opportunities, and lessons learned. Notably, up to 40% and 22% of total COVID-19 PCRs done in Sindh in the first 2 months of the pandemic, respectively, were performed at Indus Hospital in Karachi, though this percentage declined gradually as other centers caught up with their testing capacities. The rapid scaling up was achieved through a combination of mechanisms and factors including building on preexisting partnerships between the GoS and IHHN, pooling resources and harnessing distinct and complementary roles, relocating existing resources, introducing automation and information technology system changes, establishing risk mitigation strategies, and introducing quality measures within testing processes. The primary outcome of the partnership was rapid province-wide access to quality COVID-19 PCR testing with short turnaround times and at no cost to the patient. Furthermore, implementation of the partnership goals established new mechanisms as well as strengthened existing ones to enable rapid response to the future global health security challenges in Sindh, Pakistan.

## Abstract (precis)

Building upon an existing public-private partnership enabled the rapid and effective implementation of province-wide COVID-19 testing in the Sindh province of Pakistan.

## Key Findings

- The outcomes of the public-private partnership between the Government of Sindh and a private organization include improving access to free of cost, scaling-up access to high-quality coronavirus disease (COVID-19) testing with rapid turnaround times in many underserved areas of Sindh, establishing and improving testing systems, and creating a mechanism of coordination and communication.
- Implementation strategies of such a partnership involve having distinct and complementary roles by both parties, effectively using existing resources, taking concrete steps to improve efficiencies, establishing risk mitigation strategies, introducing measures of quality and safety within testing processes, enabling rapid coordination and communication, as well as ensuring the commitment of the management and staff to handle the crises.

## Key Implications

- Governments should consider pooling resources through effective public-private partnerships to enable rapid implementation of responses to global health security challenges such as that posed by COVID-19 PCR testing crises at the beginning of the pandemic.
- The successful implementation of the partnership goals provides a model that can be replicated and emulated to enable rapid and effective responses to future global health security challenges in Pakistan and other low- and middle-income countries.

## INTRODUCTION

A 2017 United Nations Development Programme report on Pakistan’s human development^1^ ranks Sindh, a province of 47.9 million,^2^ as second among Pakistan’s provinces in terms of human development index—a composite index of health and standard of living of a population— but with the largest intraprovince variation. The report notes that districts outside the megacities of Karachi and Hyderabad, where 65% of the population of Sindh resides,^3^ are among the worst-performing in Pakistan in terms of the human development index. This translates to gross structural and operational inadequacies of the health care system across much of Sindh and sparse to nonexistent polymerase chain reaction (PCR) testing facilities at the start of the coronavirus disease (COVID-19) pandemic. Against this backdrop and exigency of the pandemic, the Government of Sindh (GoS) initiated a public-private partnership to improve access to COVID-19 PCR testing across the Sindh province.

The collective leadership of the partnership envisaged a rapid scaling up of province-wide, high-quality COVID-19 PCR testing with short turnaround times and at no cost to the patient. We share the rapid scaling up and other implementation strategies used in the first 6 months, starting in March 2020, when the partnership for COVID-19 PCRs in Sindh began. We also discuss the challenges and lessons learned during this process. Overall, the partnership has been implemented and evaluated for 1 year, as reflected in some of the summarized data we present.

The collective leadership of the partnership envisaged a rapid scaling up of province-wide, high-quality COVID-19 PCR testing with short turnaround times and at no cost to the patient.

## THE PUBLIC-PRIVATE PARTNERSHIP

The partnership was initiated without a formal agreement being signed between the GoS and the Indus Hospital & Health Network (IHHN), the private nonprofit organization of the partnership (Box). This was done not only because of time constraints but also because the pandemonium of the time meant that circumstances were changing rapidly and formal agreements were deemed a hindrance to the much-needed rapidity of planning and flexibility of actions. Instead, a COVID-19 Response Task Force was constituted that included representatives from both partners, other health care delivery public and private organizations, and civil society members including philanthropists and nongovernmental organizations. The Task Force had the mandate to steer the partnership’s response, in addition to being an attestation body for any decision mutually undertaken by both partners.

BOX **Indus Hospital & Health Network (IHHN): The Private, Nonprofit Organization of the Public-Private Partnership (PPP)**

IHHN’s overarching mission and founding principle is to provide quality health care at no cost to the patient while adopting a multifront approach for addressing the preventive, primary, secondary, tertiary, and rehabilitative health care needs of the people of Pakistan (Supplement). The infrastructure, programs, and operations of IHHN are funded by several partners including government, private donors, and international organizations but directed and managed entirely by IHHN.

Importantly, all of IHHN’s institutions and programs comply with its overarching mission of providing health care free of cost to people. IHHN manages 13 tertiary care hospitals, 8 of which are funded by the government and 5 are funded by philanthropic private donors and organizations, as well as other local and international donors.

Indus Hospital at Karachi, one of the 5 privately funded hospitals, participated in this PPP. In addition, the Institute is the location of IHHN’s headquarters and is its first and oldest institution. Some of IHHN’s key public health programs, of a countrywide scope, in collaboration with the government and other partners, include control of TB, malaria, hepatitis C, hepatitis B, HIV, and rabies.

IHHN’s other preexisting and effectively implemented partnerships with the government and its noncommercial, free of charge, health care delivery model enabled the requisite trust needed to urgently initiate and establish coronavirus disease (COVID-19) PCR testing under a PPP.

Under the understanding reached between both partners through the COVID-19 Response Task Force, the GoS district health offices (DHOs) from across Sindh administered sample collection and transport to Indus Hospital at Karachi where PCR testing was centralized. Similarly, the GoS covered the costs of testing kits while Indus Hospital at Karachi covered other consumables, overheads, and human resource costs.

Since time was a critical metric for the partnership goals, the situation was declared to be an exceptional emergency by the GoS, allowing for suspension and bypassing of bureaucratic procedures that did not directly impact the goals of the partnership. For example, to handle procuring the requisite reagents and consumables, funds were transferred by the GoS to Indus Hospital at Karachi through grant-in-aid. This was a mechanism for legally circumventing the lengthy government procurement procedures. Through these grant-in-aid funds, procurement was directly handled by Indus Hospital at Karachi.

## RESULTS

Notwithstanding the challenges, as detailed in the following sections, the most notable outcome of the partnership’s response was the swiftness with which mechanisms were established and executed to scale up COVID-19 PCR testing on samples collected from across the province.

The most notable outcome of the partnership’s response was the swiftness with which mechanisms were established and executed to scale up COVID-19 PCR testing on samples collected from across the province.

**FIGURE 1:**
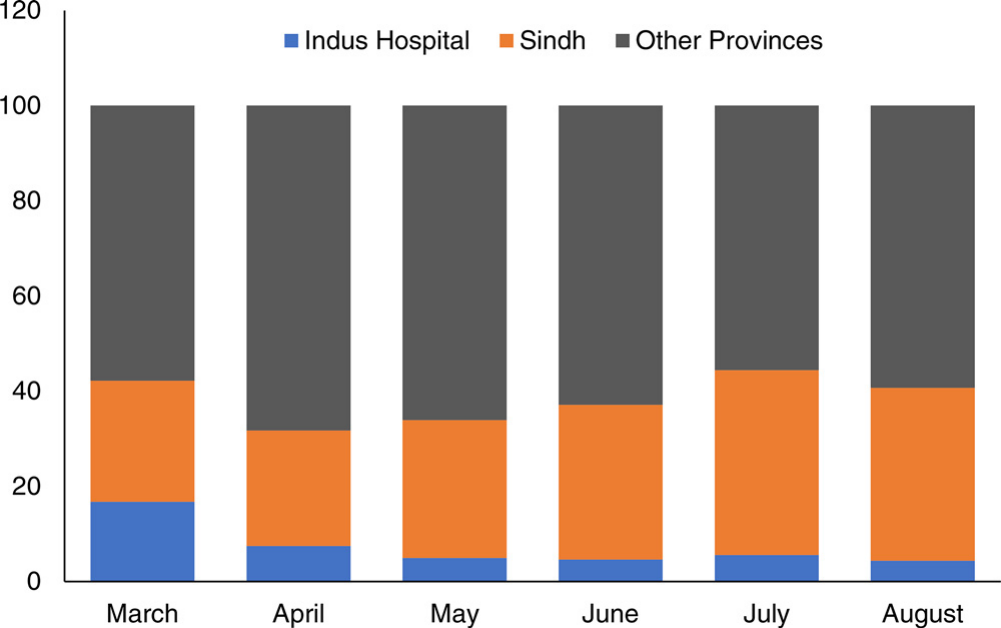
Monthly Percentages of COVID-19 PCR Tests Conducted in Pakistan^a^ Abbreviations: COVID; coronavirus disease; PCR, polymerase chain reaction. ^a^Performed at Indus Hospital, Karachi, the rest of Sindh, and other provinces of Pakistan respectively from March 2020 to August 2020.

In the first 6 months of the pandemic, approximately 2.6 million^4^ PCR tests for COVID-19^4^ had been performed in Pakistan at 156 testing centers,^5^ 39% (approximately 1 million) of which were conducted in Sindh.^5^ Of those conducted in Sindh, around 130,000 were performed at Indus Hospital at Karachi, approximating to 13% of province-wide, and 5% of the total testing in Pakistan during this period (Figure 1).

**FIGURE 2:**
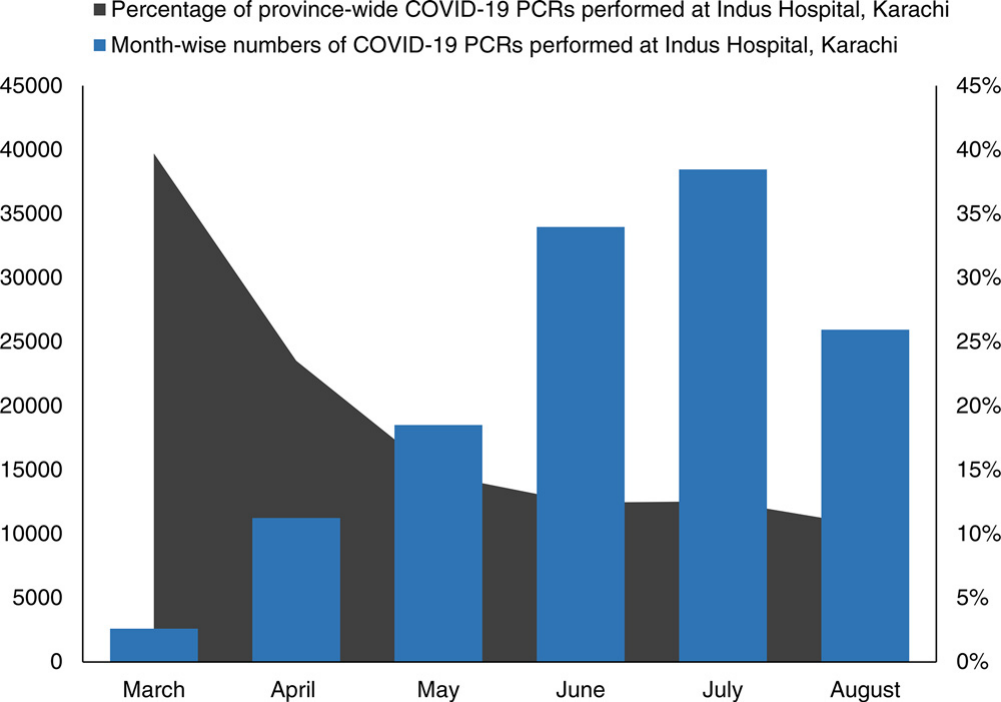
Monthly Total of COVID-19 PCR Tests Conducted in Sindh, Pakistan^a^ Abbreviations: COVID; coronavirus disease; PCR, polymerase chain reaction. ^a^Performed at Indus Hospital, Karachi, from March to August 2020. The dip in August in the total number of tests done is due to torrential rains that interrupted sample collection and transport from many remote sites of Sindh.

Notably, up to 40% and 22% of total COVID-19 PCRs done in Sindh in the first 2 months, respectively, were carried out through the GoS-IHHN partnership (Figure 2). While the monthly test numbers at Indus Hospital, Karachi, showed an upward trend, the percentage of total tests conducted in Sindh steadily decreased in the first 6 months, as other centers in Sindh, 39 in total,^5^ gradually caught up with capacities to conduct COVID-19 PCR testing.

## COVID-19 PCR TESTING SCALE-UP CHALLENGES AND SOLUTIONS

### Procurement of Consumables and Supplies

#### Alternative Mechanisms for Assessing Quality of Testing Kits

As normal procurement rules, with inbuilt mechanisms of quality control, were temporarily suspended, alternate ways of quality assessment of the available testing kits needed to be established. A GoS purchase committee comprising technical and managerial staff from both the private and public sectors was constituted. The committee’s mandate was to search for the availability in Pakistan of COVID-19 PCR testing options, contact suppliers, review all available information on test reliability, assess the feasibility of each test including the market availability of associated supplies and consumables and compatibility with existing machines, and make recommendations regarding the procurement of the kits. All kits and other reagents and consumables approved by the purchase committee were also validated at Indus Hospital, Karachi according to U.S. Food and Drug Administration guidelines.^6^

At the outset, such validations needed to be done frequently because supply chain issues often hindered the uninterrupted supply of a type of kit or other consumables.

#### Shortage of Testing Kits and Other Consumables

An acute shortage of testing kits and other requisite consumables (e.g., swabs for sampling and viral transport media) was another challenge to keep up with the demand for COVID-19 PCR testing. Since the positivity rate for COVID-19 was low in the initial months of the pandemic, a protocol for “pool testing” was developed and validated. This allowed the pooling of up to 7 samples in a single test, increasing the testing capacity without additional resources by several-fold. Any positive result in a pooled test would be followed up by retesting separately all the samples constituting the pool. With more streamlined supply chain mechanisms and because of the rapidly increasing positivity rate, pool testing was soon archived.

Since the positivity rate for COVID-19 was low in the initial months of the pandemic, a protocol for “pool testing” was developed and validated.

For some other essential consumables, urgent plans were made for local manufacturing. Viral transport media were prepared in-house at Indus Hospital, Karachi following World Health Organization guidelines, ^7^ while the GoS organized the manufacturing of sampling swabs locally.

### Sample Collection

#### Sample Collection Outreach

**FIGURE 3:**
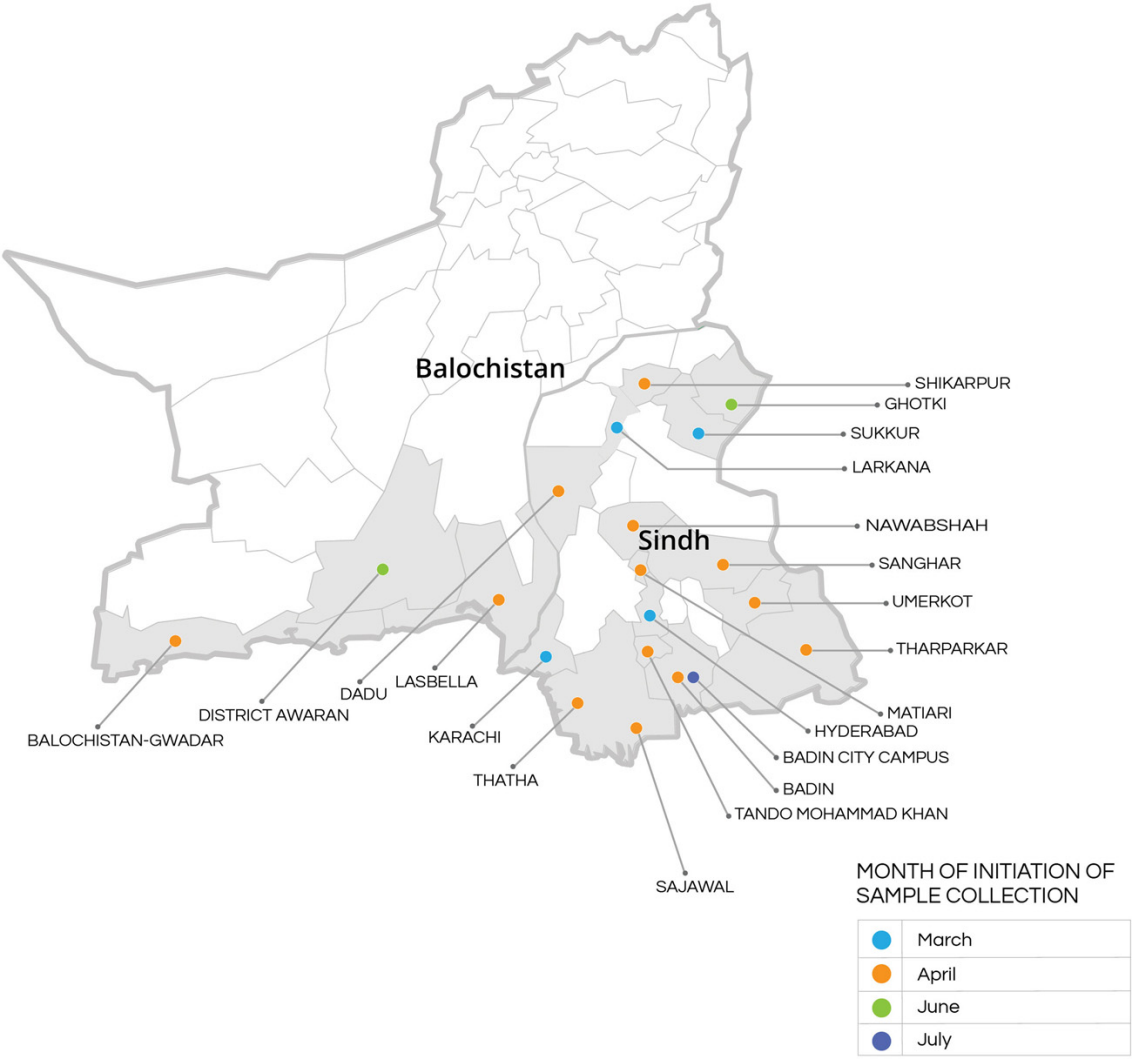
COVID-19 PCR Testing Outreach of Government of Sindh and IHHN Partnership Abbreviations: COVID; coronavirus disease; IHHN, Indus Hospital & Health Network; PCR, polymerase chain reaction.

The partnership's COVID-19 PCR testing outreach extends across Sindh and part of Balochistan (Figure 3). Overall, 70% of the total sample collection in the first year of the partnership was from the sites operated by the GoS, while 30% of samples were collected from sites operated by IHHN. GoS sample collection sites included those located inside hospitals for patients, as well as in the community for screening and contact tracing. Each GoS DHO coordinated sample collection from their respective districts. Similarly, IHHN collected samples from the community through its TB community screening infrastructure, as well as from some of IHHN’s hospital-based sites.

#### Shortage of Trained Staff for Sample Collection

At the outset, there was an acute shortage of trained staff to establish and run the extensive sample collection network, especially because most sample collection at the beginning of the pandemic was done at homes of potentially infected individuals and their contacts. This was initially managed by the GoS through urgent training and deployment of the government door-to-door polio immunization program staff for COVID-19 PCR home sampling. Moreover, all available staff in each district under the DHOs, including laboratory technicians and dispensers, were trained for swabbing and sample collection for COVID-19 PCRs. Within a few weeks, as the polio immunization program resumed, staff nurses were hired on a contractual basis and trained in sample collection at Indus Hospital, Karachi among other centers.

#### Coordination of Sample Collection Activities

A district control room was established with the mandate to coordinate the pandemic-related activities in each district. These control rooms received and compiled lists of home screenings from various sources, including the district surveillance office, that needed to be done in each district and dispatched the lists to the DHOs.

#### Quality Control of Sample Collection

**FIGURE 4:**
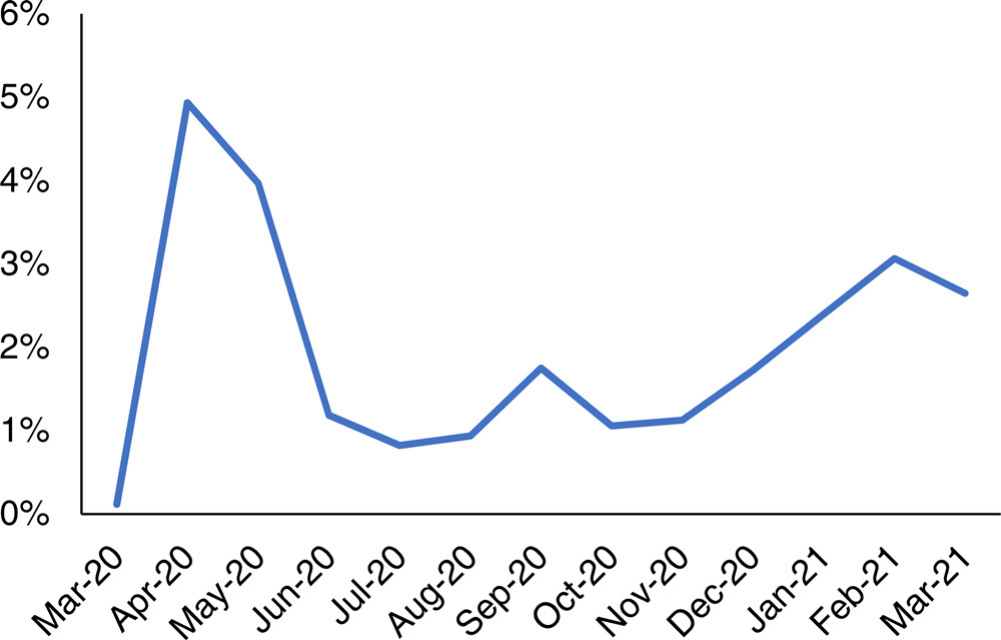
Month-Wise Sample Rejection Data of COVID-19 PCR Tests in Sindh, Pakistan, From March 2020–March 2021 Abbreviations: COVID; coronavirus disease; PCR, polymerase chain reaction.

Indus Hospital at Karachi defined sample acceptance criteria according to international guidelines and communicated the criteria to all collection sites. The sample rejection percentage of the samples that did not meet the acceptance criteria was monitored in the laboratory. This monitoring was supplemented by the laboratory conducting ongoing training and communication with sample collection and transport staff about sample acceptance standards. Sample rejection was relatively high at the outset but declined gradually as staff got more trained and the process streamlined (Figure 4).

Sample rejection was relatively high at the outset but declined gradually as staff got more trained and the process streamlined.

### Sample Storage, Transport, Cold Chain Maintenance

Cold chain maintenance and availability of transport facilities for samples—from the time of collection to transport and temporary storage at DHOs, and transport to Indus Hospital, Karachi—were other major challenges at the start of the pandemic. This crisis was handled through the temporary suspension of the government Expanded Programme on Immunization and relocation of the program’s resources including cold chain maintenance equipment (storage boxes, ice packs, and refrigerators), staff, and transport facilities (vehicles and drivers) for COVID-19 testing-related activities. In the ensuing few weeks, the Expanded Programme on Immunization services resumed and the relocated machinery was replaced by dedicated ones for COVID-19 cold chain maintenance and transport.

### Laboratory Testing: Capacity, Platforms, and Systems Development

The province-wide scope of the program and its rapid escalation also needed efficient laboratory systems and processes. At the outset, this was hindered by a lack of training and manual methods of testing and documentation. Broadly, PCR involves 2 major procedures: nucleic acid extraction and amplification. COVID-19 PCR testing was initiated using manual methods because automated options were unavailable for COVID-19 testing.

**FIGURE 5:**
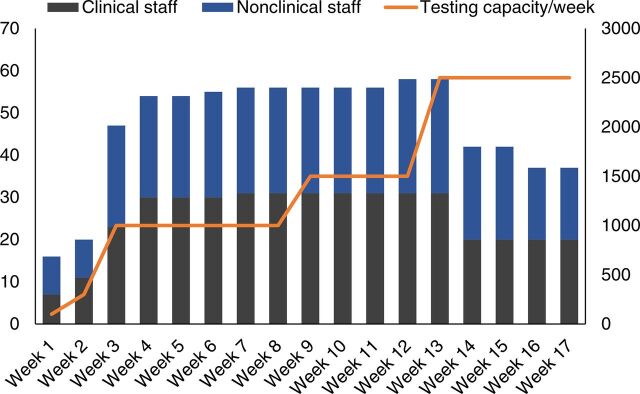
Weekly Workloads and Staff Numbers at the COVID-19 PCR Laboratory at Indus Hospital, Karachi, Pakistan Abbreviations: COVID; coronavirus disease; PCR, polymerase chain reaction.

At first, the existing electronic hospital management information system was upgraded with a new test identification number for COVID-19 PCRs. In addition, the laboratory changed to operate 24 hours a day to maximize the use of the available space and equipment and for samples collected across Sindh to be received and processed at any time they arrived, which was often in the late evening or night from remote sites. The existing systems and procedures allowed for a testing capacity of up to 700 tests in week 1 (March 16–21, 2020). As sample influx continually increased, the throughput achievable with these existing resources and techniques reached its limit. A 3-fold enhancement of this capacity to 2,100 tests the following week (Figure 5) was achieved by introducing electronic systems, including electronic test requisition, electronic generation of medical record numbers, barcode printer-generated investigation request slip numbers for sample tubes, tagging of PCR plates and report sheets with barcodes, and barcode scanning replacing manual data entry.

A 3-fold enhancement of testing capacity was achieved by introducing electronic systems.

Without dedicated COVID-19 PCR testing kits for automated equipment at hand, hepatitis C virus (HCV) nucleic acid extraction kits, already in use with Abbott m-2000sp as part of IHHN’s HCV eradication program, were validated and repurposed for severe acute respiratory syndrome coronavirus 2 (SARS-CoV-2) nucleic acid extraction. From March 30 to April 6, 2020, fully automated SARS-CoV-2 nucleic acid extraction was initiated, enhancing the lab's capacity to conduct 7,000 tests a week.

With the availability of GeneXpert cartridges in Pakistan in week 11 (May 25–31, 2020) the laboratory was able to expand its testing options by utilizing the GeneXpert machines already in use for the existing TB control program. GeneXpert is a PCR testing system that returns results in less than an hour. This not only helped increase testing capacity from 7,000 tests a week to 10,500 but also improved emergency patient care. However, because the supply of these cartridges was limited, IHHN restricted testing with GeneXpert to emergency department patients requiring urgent procedures or admissions to the intensive care unit.

With the availability of COVID-19 PCR kits compatible with automated systems in week 15 (June 15–21), fully automated testing using Roche Cobas 6800 and Abbott M2000sp and M2000rp was initiated. These instruments were also interfaced with the existing electronic laboratory information system, thus obviating the need for manual results entry and further improving efficiency. The following week, electronic patient data entry in some of the outreach sites was initiated.

By the end of March 2021, the laboratory had a testing capacity of 24,500 tests per week and had conducted more than 400,000 COVID-19 PCRs. However, systems at all the outreach sites could not be transitioned to electronic because of the shortage of data entry staff and other electronic equipment needed. This would have credibly increased the efficiency of the systems further.

Some of the risks to the laboratory’s smooth operations included the frequent, yet random power and internet outages, supply chain issues relating to testing kits and other consumables, and safe handling of the excessive waste generated. These were addressed through providing backup power supplies, using wireless radio links and backup internet devices, installing multiple platforms and testing methods to counteract supply chain issues and instrument breakdowns, as well as procuring additional autoclave systems to enable safe management of additional waste.

### Quality Assurance

Quality assurance mechanisms needed to be instituted to measure the reliability of results. These mechanisms included the development of and training in standard operating procedures covering all phases of COVID-19 PCR testing, including sample collection, transport, PCR testing, generation, and dispersion of results. These also included defining, benchmarking, and evaluating relevant and easily retrievable performance indicators. Evaluation of these indicators at regular intervals was followed by root cause analysis, corrective, and preventive actions in cases of any deviation from established benchmarks.

**FIGURE 6:**
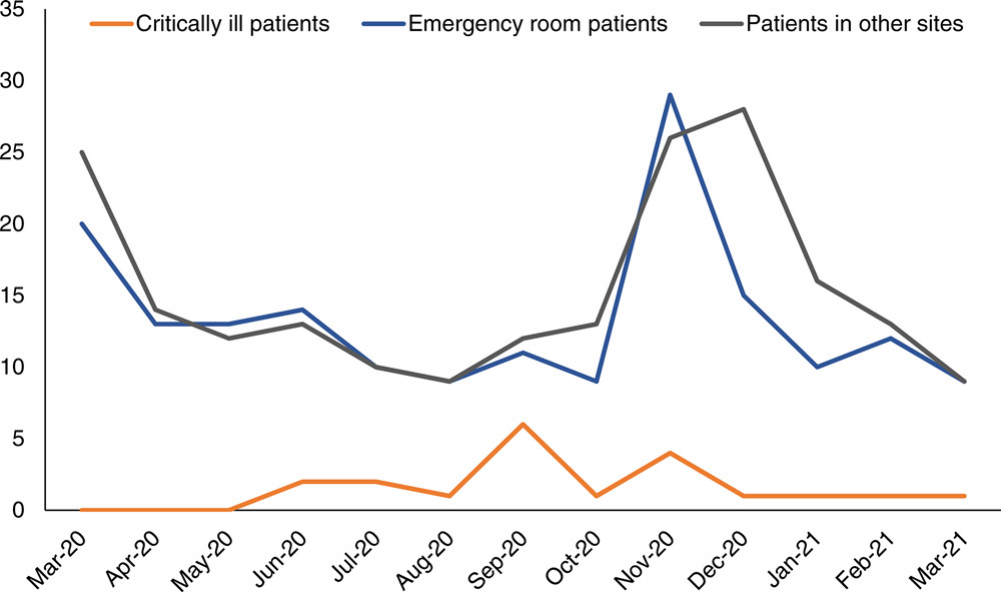
COVID-19 PCR Result Turnaround Times for Samples Received From Different Sites in Sindh, Pakistan^a^ Abbreviations: COVID; coronavirus disease; PCR, polymerase chain reaction. ^a^From March to August 2020, the turnaround times improved because of a gradual introduction of automation. From September to December 2020, times started to show an upward trend because of a gradually increasing workload of samples from remote sites for which electronic patient data entry was not an option. These times gradually reduced thereafter as more data entry staff were hired at Indus Hospital’s PCR lab to cater to the increasing workload.

Turnaround time was benchmarked at 24 hours for COVID-19 PCR samples received from nonemergency and screening sites (Figure 6), while for samples collected in hospital emergency rooms, most testing and reporting were completed within 12 hours. For critically ill patients, the target turnaround time was reduced to 2 hours with the availability of GeneXpert SARS-CoV-2 cartridges.

**FIGURE 7:**
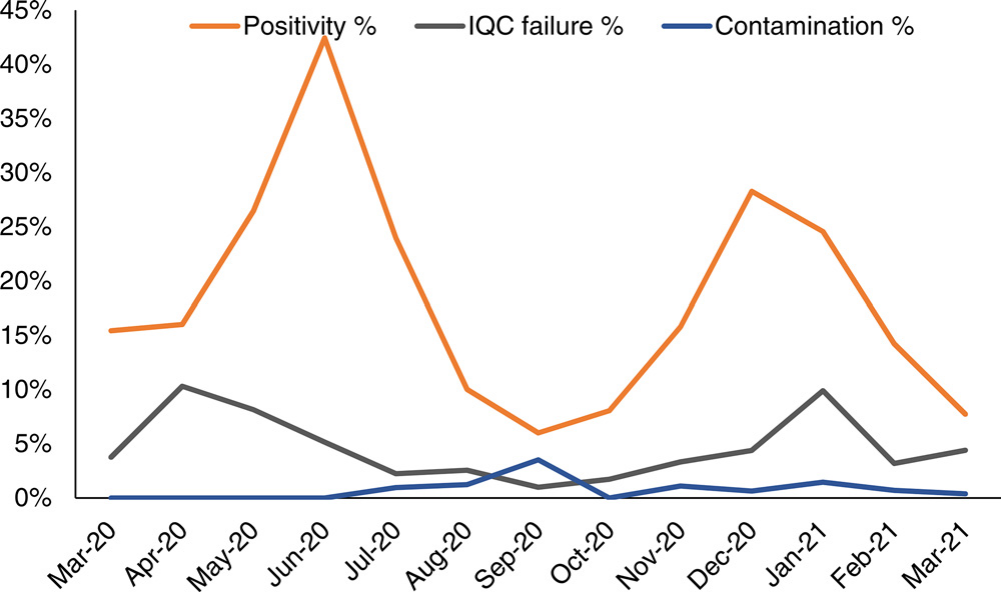
Percentage of Positive Results, Internal Quality Control Failures, and Contamination After COVID-19 PCR Tests at Indus Hospital, Karachi, Pakistan, From March 2020–March 2021^a^ Abbreviations: COVID; coronavirus disease; IQC, internal quality control; PCR, polymerase chain reaction. ^a^The spike in January 2021 in the percentage of internal control failures is due to a shortage of automated testing kits and the use of a new, previously unused manual COVID-19 PCR testing kit supplied by the Government of Sindh. The kit was subsequently not used due to suboptimal performance.

Other performance indicators used for monitoring were the percentage of samples rejected (Figure 5) and tests that needed to be repeated (Figure 7). Repeat testing key performance indicators included 2 kinds of quality control failures: contamination and internal quality control failures.

Additional quality assurance measures included participating in both local (National Institute of Health, Pakistan) and the College of American Pathologists SARS-CoV-2 proficiency testing programs (Supplement), conducting method comparison validation studies before selecting instruments and kits, and complementing staff training with competency assessments for each task. Moreover, a quality management system under International Organization for Standardization standards was implemented for COVID-19 PCRs.

### Laboratory Personnel Management

#### Staff Relocation

Many staff, including pathologists, managers, technologists, and other nonclinical support staff from all clinical laboratory sections, were relocated to the nascent COVID-19 PCR laboratory. This was initially possible because workloads declined in other sections as a consequence of the lockdown beginning March 2020. However, as the workload increased toward the first week of June 2020, the efficiency of the COVID-19 laboratory also improved by introducing electronic systems, staff training, and automation, so that some staff could revert their respective departments even as the COVID-19 PCR testing workloads increased (Figure 5).

The COVID-19 laboratory efficiency improved by introducing electronic systems, staff training, and automation, so that some staff could revert to their respective departments even as the COVID-19 PCR testing workloads increased.

#### Training

Before the pandemic, Indus Hospital, Karachi had limited experience with manual PCRs because they had only one trained staff person available to perform the procedure. Thus, the deployment of staff for the COVID-19 PCR laboratory and their training needed to be undertaken on an emergency basis.

To train the upward of 50 staff relocated for COVID-19 PCRs, assistance from other public sector institutions was mediated by the GoS, under which staff from a government institution with experience in manual PCRs assisted with onsite training of the COVID-19 PCR laboratory staff of Indus Hospital, Karachi. Trainers demonstrated techniques and then directly observed staff undergoing training. Competence assessment sheets were developed, which were used to document the step-by-step competence in performing PCR procedures. PCR-interpretation training was undertaken through crossed-verification of results by trained staff. Credentialing for independent reporting was completed following 200 consecutively concordant results of trainees and trainers.

SARS-CoV-2 is a Biosafety Level 3 (BSL-3) virus, requiring specialized facilities and training in handling samples. A BSL-3 facility already existed at Indus Hospital, Karachi, and training in specialized BSL-3 protocols was thus organized at the existing facilities. For continued staff training and supervision, WhatsApp group messaging was also used. Through these groups, noncompliances with established protocols were notified, problems addressed, technical questions answered, and directions given.

#### Staff Morale

To keep up staff morale and acknowledge their services during those trying circumstances, a COVID-19 frontline worker daily allowance was added to their salaries. Other concrete steps in this direction included providing door-to-door transport facilities and free meal and snack services in addition to direct and frequent employee appreciation and motivation from senior management, which maintained an onsite presence during all shifts.

#### Coordination and Communication

A coordination committee including members from GoS and IHHN, as well as civil society members including philanthropists, other nongovernmental organizations, private sector entities, and pathologists, was set up to facilitate coordination between the diverse group of stakeholders involved in operating the program. To address daily operational challenges, WhatsApp instant messaging groups were created as telecommunication services are widely available across Pakistan,^8^ but internet and email access is a challenge across much of Sindh.

WhatsApp groups were also used to share with all relevant stakeholders data needed for planning purposes. These were generated from the laboratory’s electronic system and included COVID-19 positivity rate, sample numbers received, daily throughput, the number of pending tests, and inventory records.

Shift In-Charge Over Forms were designed for seamless transition of operational information including the number and location of all in-process samples and site-wise sample numbers received.

### Result Dispersion

Maintaining rapid result turnaround times without electronic systems to disburse PCR results across much of Sindh was another major challenge. To shorten the disbursement time, an automated system of instant phone messaging to patients indicating their status as COVID-19 “positive” or “negative” was created, thus potentially allowing for rapid action before the detailed report was delivered. In addition, reports in PDF format were also emailed daily to the respective DHOs, which were able to send those results to individuals through WhatsApp messaging. DHOs also provided information on how to access the results directly from IHHN’s website.

To shorten the result disbursement time, an automated system of instant phone messaging patients with their result status was created.

Aggregate data were also sent to the GoS in the prescribed format to align with the government requirements, which were eventually pooled in a centralized database by the GoS. In addition, the names, addresses, and national ID card numbers of all positive cases were also sent to the health authorities for contact tracing and quarantining.

## LESSONS LEARNED

We detail some key lessons learned by implementing the partnership for rapid scale-up of COVID-19 PCR testing in Sindh.

### Build Upon Existing Public-Private Partnerships to Enable Fast Response

Since a critical factor for the success of the partnership goals was time, some of the measures hastily undertaken would not have been possible in a short amount of time without the preexisting and effectively implemented partnerships between GoS and IHHN. Building upon the existing partnership enabled fast action without the need to wait for signed agreements or procurement procedures. In addition, both partners aligned on the overarching goals, namely to deliver public health care for noncommercial interests.

### Harness the Distinct Strengths of Each Partner and Pool Resources of the Partnership

The GoS and IHHN had distinct and complementary roles that allowed both partners to contribute and pool resources that did not overlap. The GoS had the DHO infrastructure that extended across the entire province that was used for sample collection. Indus Hospital, Karachi had the resources for laboratory-based testing. Harnessing these assets and strengths allowed both partners to contribute to the program’s success as equal partners and for the credit and responsibilities to be equally shared.

Harnessing the assets and strengths allowed both partners to contribute to the program’s success as equal partners and for the credit and responsibilities to be equally shared.

### Relocate Existing Resources for a Rapid Response

GoS relocated staff and other equipment from existing programs, including the Expanded Programme on Immunization and polio door-to-door immunization, to be used for COVID-19 sampling and transport.

Similarly, IHHN repurposed staff, facilities, and resources from the preexisting public health programs, including TB and HCV infection, for COVID-19 testing. The TB screening and surveillance system was used for community-based SARS-CoV-2 sampling, while BSL-3 facilities were used for PCRs. GeneXpert machines, already in place for TB testing, provided supplemental COVID-19 PCRs for critically ill patients, while preexisting Abbott M2000sp machines and HCV nucleic acid extraction kits were repurposed for automated SARS-CoV-2 nucleic acid extractions.

Although gradual resumption of all those programs followed, the initial relocation bought time for immediate implementation of the COVID-19 testing crisis response.

### Use Messaging Apps to Rapidly Communicate and Coordinate

The massive COVID-19 testing program could not have been implemented without effective communication using WhatsApp group messaging for training, sharing data, and dispersing reports. Using a messaging platform for group-based messaging can make communication more efficient.

## CONCLUSION

With the limitations that the global shortage of laboratory consumables posed, the key indicators of the success of the partnership between GoS and IHHN were rapid scaling-up of province-wide access to high-quality COVID-19 testing with rapid turnaround times and at no cost to the patient.

Since March 2021, when the first year of the public-private partnership concluded, most of the COVID-19 PCR testing has gradually shifted to the GoS facilities that have increased their testing capacity. The lessons learned and the mechanisms established through this partnership have increased the global health security capabilities of the Sindh province. This is credibly expected to further reduce the lag time for mounting a coordinated, rapid, and effective response in Sindh to future global health security challenges.

## Supplementary Material

21-00308-Aijaz-Supplement.pdf

## Notes

**Peer Reviewed**

**First published online:** February 9, 2022.
