# Supplementary material for: COVID-19 Testing Crisis Management Through a Public-Private Partnership in Sindh, Pakistan
Source: Glob Health Sci Pract. 2022 Feb 28;10(1):e2100308. doi: 10.9745/GHSP-D-21-00308 (PMC8885349; doi:10.9745/GHSP-D-21-00308)
Supplement: 21-00308-Aijaz-Supplement.pdf [file 21-00308-Aijaz-Supplement.pdf]

Supplement to: Jamal S, Aijaz J, Shah N, et al. COVID-19 testing crisis management through a public-private Partnership in Sindh, Pakistan. *Glob Health Sci Pract*. 2022;10(1):21-00308. https://doi.org/10.9745/GHSP-D-21-00308

Supplement Figure. Outreach of Health Care Services Provided by Indus Hospital & Health Network, the Private, Non-Profit Organization of the Public-Private Partnership

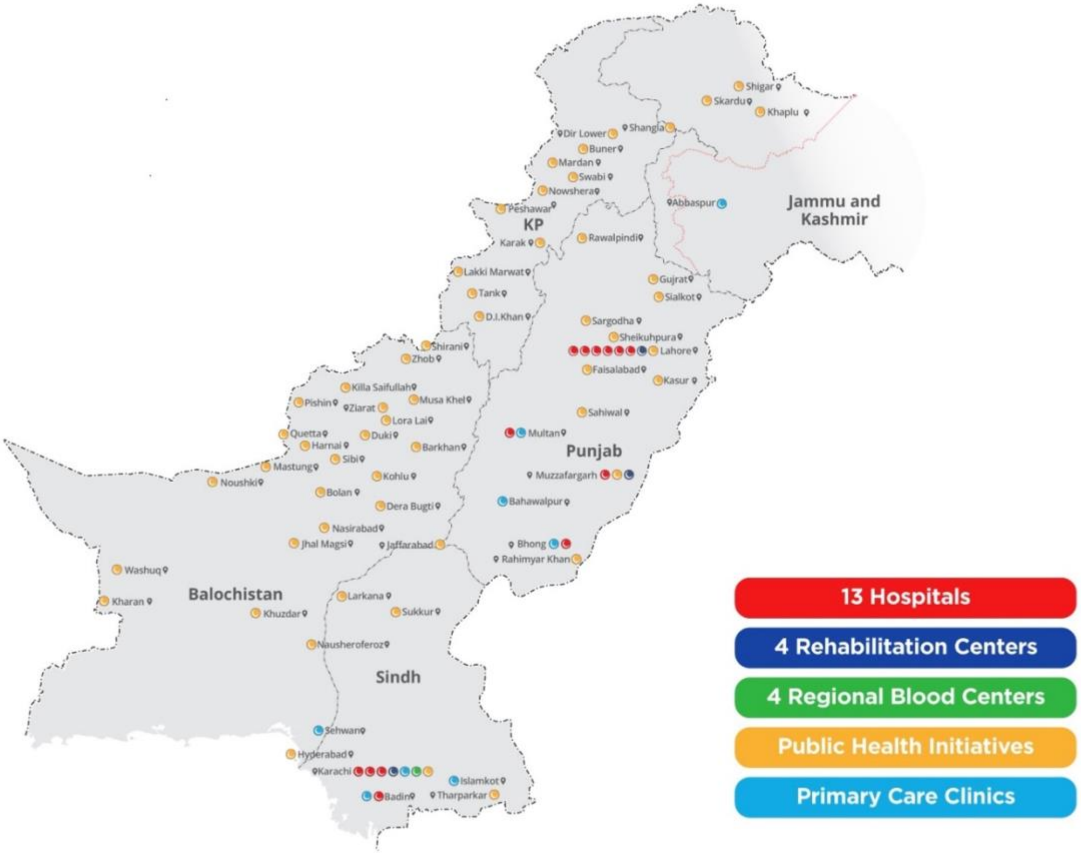

Supplement to: Jamal S, Aijaz J, Shah N, et al. COVID-19 testing crisis management through a public-private Partnership in Sindh, Pakistan. *Glob Health Sci Pract*. 2022;10(1):21-00308. https://doi.org/10.9745/GHSP-D-21-00308

Supplement to: Jamal S, Aijaz J, Shah N, et al. COVID-19 testing crisis management through a public-private Partnership in Sindh, Pakistan. *Glob Health Sci Pract*. 2022;10(1):21-00308. https://doi.org/10.9745/GHSP-D-21-00308

Supplement Tables. Quality Assurance Measures Used in COVID-19 PCR Testing at Indus Hospital, Karachi

College of American Pathologists (CAP) Proficiency Testing Survey		
**Method**	**Cycle (Month)**	**Comparison with participant summary**
Roche Cobas 6800/8800	COV2R-A 2020 (July-20)	Sample 1: 100%
		Sample 2: 100%
		Sample 3: 100%
	COV2R-B 2020 (Dec -20)	Sample 1: 99%
		Sample 2: 98%
		Sample 3: 98%
	COV2-A 2021 (May-21)	Sample 1: 100%
		Sample 2: 100%
		Sample 3: 100%

Educational Quality and Assessment Programme (EQAP) by National institute of Health (NIH)		
**Method**	**Cycle (Month)**	**Comparison with participant summary**
Roche Cobas 6800/8800	NIH SARS CoV-2 EQAP -02 (Dec-20)	Sample 1: 100%
		Sample 2: 100%
		Sample 3: 100%
		Sample 4: 100%
		Sample 5: 100%
